# Quantification of lung collapse during peeptitration by electrical impedance tomography in experimental ards - comparison with quantitative ct analysis

**DOI:** 10.1186/2197-425X-3-S1-A995

**Published:** 2015-10-01

**Authors:** S Hammermüller, E Costa, M Amato, K Noreikat, W Brehm, S Wolf, UX Kaisers, H Wrigge, AW Reske

**Affiliations:** Department of Anesthesiology and Intensive Care Medicine, University Leipzig, Leipzig, Germany; Faculdade de Medicina da Universidade de Sao Paulo, Sao Paulo, Brazil; Veterinary Medicine Faculty of University of Leipzig, Leipzig, Germany

## Introduction

Tidal recruitment of nonaerated lung is a main cause of ventilator associated lung injury. CT as the gold standard for quantifying lung collapse (CT-collapse) is associated with certain risks for the patient (e.g. radiation exposure or transportation) and cannot be used for repeated assessments. Electrical impedance tomography (EIT) instead is a bed-side non-invasive radiation-free continuous technique for monitoring of changes in thoracic air content and distribution. EIT may also allow quantification of recruitable lunge collapse (EIT-collapse) [1].

## Objectives

To study correlation and agreement between CT- and EIT-collapse during a decremental PEEP-titration after a lung recruitment maneuver (RM) for further validation of the technique for assessment of EIT-collapse.

## Methods

We induced ARDS in anesthetized pigs by pulmonary acid (HCl) instillation until the PaO_2_/FiO_2_ remained stable < 200 mmHg. Tidal volume was 6 ml/kg body weight. We performed a RM (PEEP 40cmH_2_O; PIP 60cmH_2_O for 2 min) followed by decremental PEEP-titration (starting from 26cmH_2_O in steps of 2 cmH_2_O). We recorded EIT-data and airway pressures simultaneously on each step and obtained end-expiratory CTs. CT-collapse in the entire lung was defined as the lung mass within -200 HU to +100 HU [2]. “Non-recruitable collapse” was defined as CT-collapse remaining after RM at PEEP = 26 cmH_2_O. Recruitable CT-collapse was calculated by multiplying the difference between CT-collapse at a certain PEEP-step and “non-recruitable collapse” by 100% and then dividing this product by the difference between total lung mass and “non-recruitable collapse”. EIT-collapse was calculated based on analysis of changes in EIT-pixel compliance [1]. The latter was estimated considering that local tidal volumes correlate well with local impedance variations. The concept used here assumes that the best compliance of a lung compartment reflects the number of functional lung units in that compartment, which, once opened, have equivalent compliances [1, 3]. Thus, the relative amount of collapse (amount of lost units) within a given pixel can be inferred from the decrease in pixel compliance in relation to its "best compliance" [1, 3]. Bland-Altman plots and within-subject linear regression were used for statistical analysis [2].

## Results

**Figure 1:**
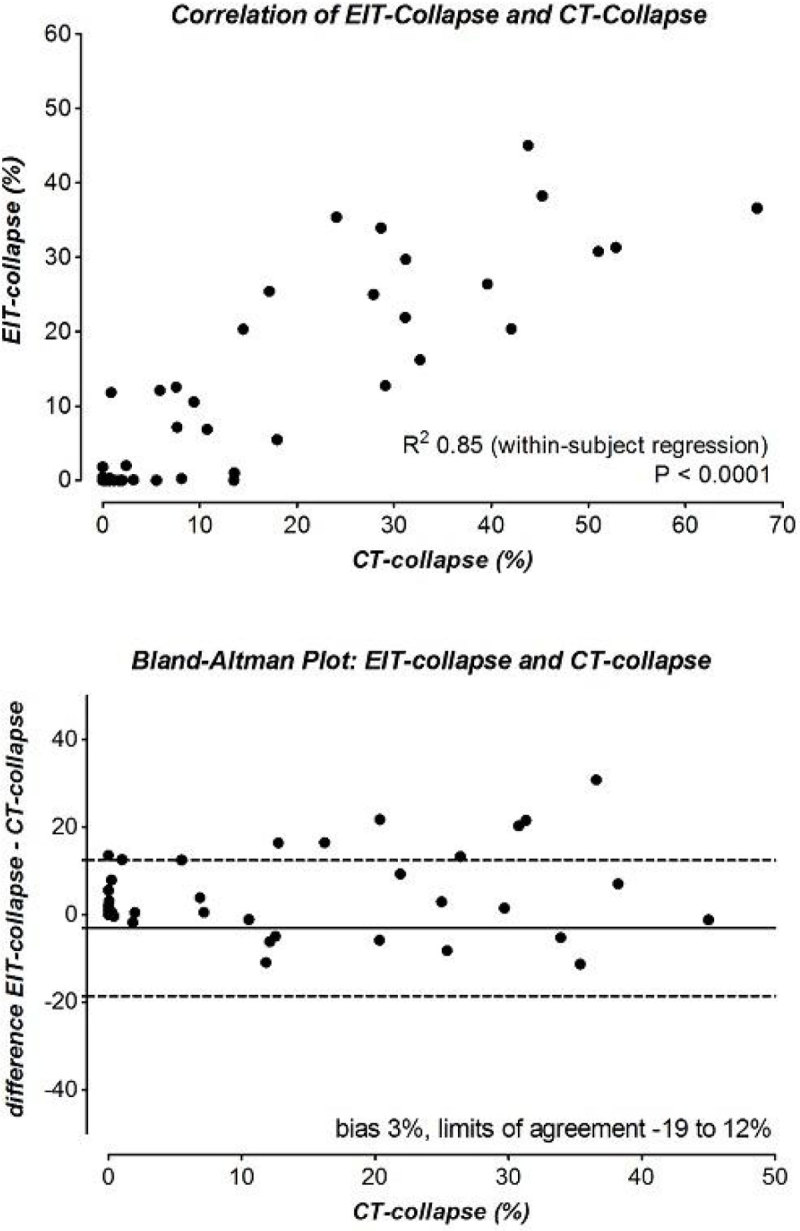
Figure 1

We analyzed 60 data points from 11 pigs (weight 39 (range 37-42) kg). We found a strong within-subject correlation and clinically acceptable agreement between CT- and EIT-collapse (Figure 1) [4].

## Conclusion

Our results support the potential of EIT for non-invasive bedside assessment of recruitable collapse.
